# Tongue-bite apparatus highlights functional innovation in a 310-million-year-old ray-finned fish

**DOI:** 10.1098/rsbl.2025.0270

**Published:** 2025-09-03

**Authors:** Sam Giles, Matthew Kolmann, Matt Friedman

**Affiliations:** ^1^ School of Geography, Earth and Environmental Sciences, University of Birmingham, Birmingham, UK; ^2^ Natural History Museum, London, UK; ^3^ Department of Biology, University of Louisville, Louisville, KY, USA; ^4^ Museum of Paleontology, University of Michigan, Ann Arbor, MI, USA; ^5^ Department of Earth and Environmental Sciences, University of Michigan, Ann Arbor, MI, USA

**Keywords:** ray finned fish, functional morphology, feeding, gill skeleton

## Abstract

Gill-skeleton modifications for processing prey represent a major source of functional innovation in living ray-finned fishes. Here we present the oldest actinopterygian tongue bite, derived from the gill skeleton, in the Middle Pennsylvanian (approx. 310 Ma) †*Platysomus parvulus*. Unrelated to extant tongue biters, this deep-bodied taxon possesses a large, multipartite basibranchial tooth plate opposing an upper tooth field centred on the vomer. This branchial structure occurs in conjunction with toothed jaws, indicating a role for both the basibranchial plate and jaws in feeding. †*P. parvulus* illustrates the assembly of the tongue bite in the geologically younger †Bobasatraniidae: large opposing dorsal (vomerine) and ventral (basibranchial) crushing plates associated with toothless jaws. The origin of tongue bites falls within the Carboniferous actinopterygian radiation, although it postdates the first signs of the consumption of hard prey (durophagy) in other ray-finned lineages by several million years. This lends support to a protracted model of actinopterygian diversification in the aftermath of the end-Devonian extinction.

## 1. Background

Living ray-finned fishes display a remarkable range of specializations associated with feeding. Among the most celebrated of these adaptations are modifications to the branchial apparatus in teleosts [1,2], including so-called ‘tongue bites’, which involve opposing dentition on the ventral surface of the braincase and palate and tooth plates on the dorsal surface of the median gill skeleton. These upper (palatal) and lower (branchial) components interact to reduce or manipulate prey [3,4]. Tongue bites and modified pharyngeal jaws serve as additional zones for prey processing, with their decoupling from mandibular jaws hypothesized to increase functional versatility [5]. Extant teleost lineages with tongue bites are relatively young, with records extending only to the Mesozoic [6]. This is a time during which feeding innovations among fishes and other predatory taxa have been implicated in major macroecological shifts in aquatic communities [7].

While the Mesozoic represents a key evolutionary interval for many extant groups, entirely extinct fish lineages show innovations that occurred independently of—and geologically long before—those in living species. Carboniferous fossils record the first episode of major morphological divergence among actinopterygians [8], apparent externally in features like body shape and dental structure [9,10]. The timing of the origin of different functional traits bears on contrasting models of actinopterygian radiation before, during and after the Devonian–Carboniferous transition. These encompass cryptic early diversification in the Devonian, a burst of innovation immediately following the boundary [11,12], and the more continuous accumulation of new ecologies throughout the Carboniferous [8]. Information bearing on the morphology of internal structures, such as the branchial skeleton, requires uncrushed specimens examined via acid preparation [13] or computed tomography [14]. The few described Carboniferous gill skeletons belong to taxa with conservative cranial anatomy [14,15] and do not differ substantially from those of Devonian examples [16].

Here, we report gill-arch structure in a uniquely three-dimensional skull of the late Carboniferous actinopterygian †*Platysomus parvulus* (‘†’ precedes extinct taxa; [17]). With its deep body and skull, weakly toothed mandibular jaws and a vertical suspensorium, †*P. parvulus* likely had distinctive locomotor and feeding ecologies relative to generalized actinopterygian conditions [11,18]. Most significantly, we find that it possesses enlarged basibranchial tooth plates that oppose a tooth field contributed to by the median vomer and paired entopterygoids. Dating to approximately 310 Ma, this represents the earliest candidate for a tongue-bite mechanism in actinopterygians, predating the oldest evidence for similar arrangements in extant groups by over 150 Myr. Modified gill-arch anatomy in †*Platysomus* amplifies the pattern of structural innovation among Carboniferous ray-fins, illustrates a step in the evolution of more elaborate tongue bites in related taxa and further highlights the widespread convergence in actinopterygian feeding strategies across clades and over time.

## 2. Material and methods

### (a) Institutional abbreviations

KUVP, University of Kansas Biodiversity Institute, Division of Vertebrate Paleontology, Lawrence, KS, USA; NHMD, Natural History Museum of Denmark, Copenhagen, Denmark; NHMUK, The Natural History Museum, London, England, UK; NMS, National Museum of Scotland, Edinburgh, Scotland, UK.

### (b) Fossil specimens

†‘Platysomidae’ *sensu* Schultze *et al.* [19]

†*Platysomus superbus*. NHMUK PV P 4060: nearly complete articulated individual in part and counterpart, Glencartholm Volcanic Beds, Upper Border Group of the Calciferous Sandstone (Mississippian: Viséan), Glencartholm, Eskdale, Dumfriesshire, Scotland, UK.

**Figure 1.**
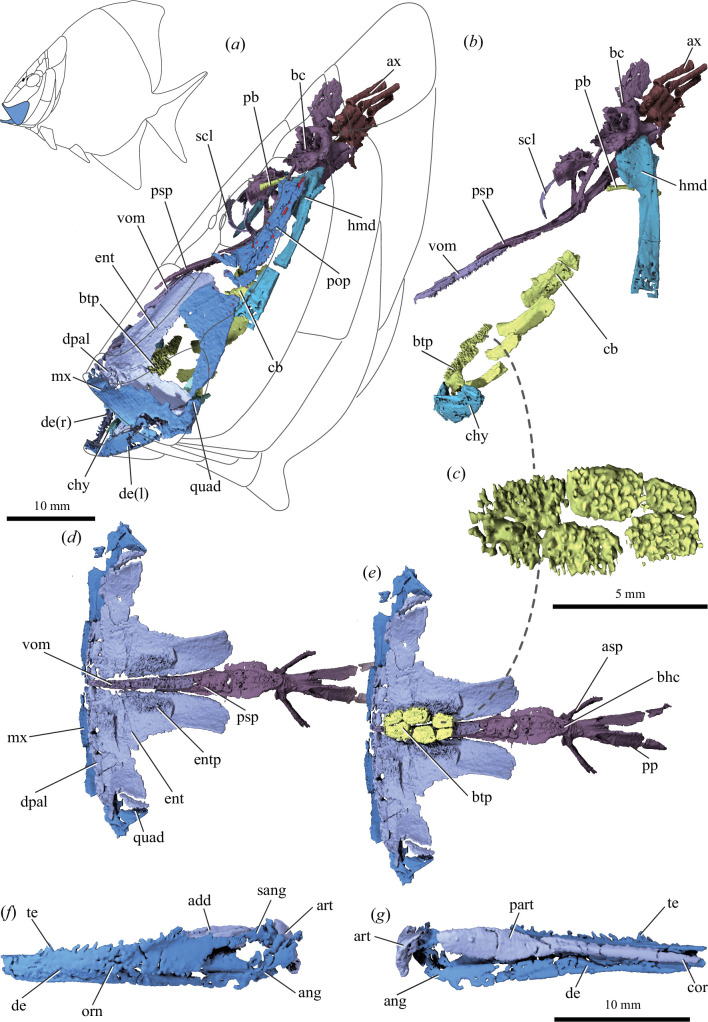
Cranial anatomy of †*Platysomus parvulus* (NHMUK PV P11697) based on µCT scanning. (*a*) Bones of skull as preserved, in left lateral view; grey lines indicate external skull bones. Inset shows reconstruction of body shape inferred from complete specimens, with maxilla and dentary in blue. (*b*) As in (*a*), but with dermal and jaw bones removed and elements repositioned. (*c*) Basibranchial tooth plate in dorsal view. (*d*) Upper jaw bones and parasphenoid in ventral view, with maxilla and palatal bones mirrored. (*e*) As in (*d*), but with basibranchial tooth plate shown in articulation. (*f*) Left mandible in lateral view and (*g*) medial view. Abbreviations: add, adductor fossa; ang, angular; art, articular; asp, ascending process of parasphenoid; ax, axial skeleton; bc, braincase; bhc, buccohypophysial canal; btp, basibranchial toothplate; cb, ceratobranchial; chy, ceratohyal; cor, coronoids; de, dentary; dpal, dermopalatine; ent, entopterygoid; entp, entopterygoid teeth; hmd, hyomandibula; mx, maxilla; orn, ornament; part, prearticular; pb, pharyngobranchial; pop, preoperculum; pp, posterior process of parasphenoid; psp, parasphenoid; quad, quadrate; sang, surangular; scl, sclerotic ossicle; te, teeth; vom, vomer. Colour coding: blue, cheek and outer jaw bones; light purple, palate and inner jaw bones; dark purple, braincase; turquoise, hyoid arch; yellow, branchial skeleton; brown, axial skeleton; red, lateral-line sensory canal pores.

**Figure 2.**
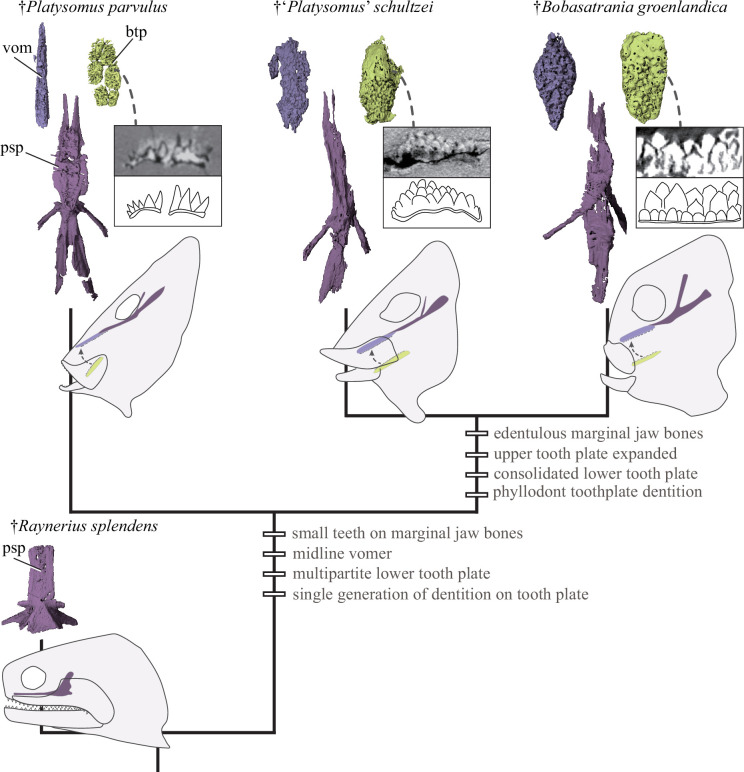
Evolution of a tongue bite in a lineage of Palaeozoic–Mesozoic actinopterygians. Upper and lower tongue-bite components in, from left to right, †*Platysomus parvulus* (NHMUK PV P11697), †‘*Platysomus*’ *schultzei* (KUVP 86168) and †*Bobasatrania groenlandica* (NHMD 161449 a); tongue-bite components absent in generalized actinopterygians (far bottom left: †*Raynerius splendens*, omitting small paired vomers; [16]). Upper row of insets show tomogram sections (upper) and schematic drawings (lower) through the lower tooth plate. Lower row of insets show schematic drawing of cranium and tongue-bite components in lateral view. Abbreviations: btp, basibranchial tooth plate; psp, parasphenoid; vom, vomer. Colour coding: light purple, upper tooth plate; dark purple, parasphenoid; yellow, branchial tooth plate.

†Bobasatraniidae *sensu* Schultze *et al.* [19]

†‘*Platysomus*’ *schultzei*. KUVP 86168: nearly complete individual in part and counterpart, Tinajas Member of the Atrasado Formation (Upper Pennsylvanian: Kasimovian), Kinney Brick Quarry, NM, USA. The parasphenoid and upper and lower tooth plates are partially exposed on the surface of the part and counterpart. The left ascending process of the parasphenoid is broken and rotated at its base such that it lies parallel to the right ascending process (figure 2; electronic supplementary material, figure S1).

†*Bobasatrania groenlandica*. NHMD 161449 a, b, nearly complete small individual in part and counterpart (figure 2; electronic supplementary material, figure S2). Specimen is heavily pyritized in some regions, and pyrite has invaded the upper and lower tooth plates, obscuring the lower portions of some phyllodont tooth cusps. NHMD 303527 a, cranial portion of a large individual. Both specimens from the Wordie Creek Formation (Lower Triassic: Induan), East Greenland.

†*Bobasatrania mahavavica*. NMS G.1956.17.15, NMS G.1956.17.16: nearly complete individual in part and counterpart from the Sakamena Group (Lower Triassic: Induan), northwestern Madagascar.

†*Platysomus parvulus*. NHMUK PV P 11697: three-dimensionally preserved head and trunk broken in multiple pieces, Knowles Ironstone, Pennine Middle Coal Measures Formation (Middle Pennsylvanian: Moscovian), Fenton, Staffordshire, England, UK (figures 1 and 2; electronic supplementary material, figure S1).

### (c) Specimen visualization

We used Nikon XT H 225 ST micro-computed tomography scanners at the Imaging and Analysis Centre, Natural History Museum, London (NHMUK PV P 11697), CoLES CT Scanning Facility, University of Birmingham (NHMD 161449 a, b and NHMD 303527 a) and CTEES Facility, Department of Earth and Environmental Sciences, University of Michigan (KUVP 86168) to visualize internal structure in specimens. Scan parameters are provided in electronic supplementary material. Materialise Mimics v. 25 (Materialise Software, Leuven, Belgium; https://www.materialise.com/en/healthcare/mimics-innovation-suite/mimics) was used to segment these data, with exported surface meshes rendered in Blender v. 2.79 (Blender Project; https://www.blender.org/). Some manual rearticulation of elements was carried out in Blender. Mandibular, hyoid and gill arch elements were realigned in †*P. parvulus* (NHMUK PV P 11697) to account for postmortem splaying of these elements and a narrow crack running across the specimen. The parasphenoid and tooth plates in †*‘P.*’ *schultzei* (KUVP 86168) are split across part and counterpart, and these were realigned; the distorted left ascending process was also restored to life position. Each slab consisted of multiple smaller blocks glued to one another; these were disarticulated and scanned separately for improved X-ray transmission.

## 3. Anatomical description

The specimen of †*P. parvulus* (NHMUK PV P 11697) consists of a skull and anterior trunk preserved in multiple pieces. Slight anteroposterior compression has flared the upper and lower jaws, making the skull appear broader than in life. Consequently, the branchial apparatus is not crushed as in a laterally compressed individual. Cracks or missing fragments mean that some bones are only preserved on one side. We focus on the feeding apparatus (figures 1 and 2; electronic supplementary material, figure S3), with other internal details intended for a subsequent study.

### (a) Palate including dorsal tooth plate and parasphenoid

A large ‘L’-shaped entopterygoid represents the principal bone of the palate (figure 1*a*,*d*,*e*). Its longer posterodorsal limb traces the profile of the parasphenoid and bears a broad, raised longitudinal band of teeth along its inner face. Four small dentigerous dermopalatines articulate in a row along the anteroventral margin of the entopterygoid (figure 1*a*,*d*). The trapezoidal ectopterygoid embraces the posteroventral limb of the entopterygoid, with a modest ectopterygoid process marking the anterior margin of the adductor chamber. The metapterygoid is unmineralized, with a small fragment of bone possibly representing a portion of the quadrate.

The parasphenoid and vomer extend along the palatal midline (figure 1*a*,*b*,*d*,*e*). Rather than being horizontal, they are oriented at a steep angle that approximates the profile of the skull. The spear-shaped median vomer is covered with a field of small, pointed teeth. When in articulation with the palate, these vomerine teeth form a continuous dental field with the longitudinal tooth bands of the entopterygoids. Tomograms show a single generation of teeth rather than superposed layers of older dentition (figure 2). The vomer inserts into a deep notch in the anterior end of the parasphenoid, posterior to which the latter broadens before sharply tapering anterior to the ascending processes. Small teeth, interrupted by a buccohypophysial opening, form a continuous band of dentition between this constriction and the vomer. There is no dermal basipterygoid process but well-developed ascending processes sweep sharply posterolaterally and extend to the skull roof. They are tube-like and completely enclose the spiracular canal along their entire length. A long posterior extension embraces the otic and occipital regions of the braincase, broadening as it approaches the occiput and split by a deep midline notch.

### (b) Hyoid arch, branchial skeleton and ventral tooth plates

The hyoid arch includes a hyomandibula, single ceratohyal and hypohyal (figure 1*a*,*b*). A small nodular endoskeletal bone near the jaw joint, located posterior to and oriented subparallel to the ceratohyal, could represent an interhyoid ossification. The hyomandibula has a broad dorsal head and long ventral shaft that is oriented vertically. Just ventral to the head, a canal for the hyomandibular trunk of the facial nerve pierces the shaft of the hyomandibula. A broad gap separates the distal tip of the hyomandibula from the single mineralized ceratohyal, which is short and plate like. The hypohyal is robust and gently curved in dorsal view.

Extensive mineralization appears limited to the ventral gill skeleton, which is most completely preserved on the left side (figure 1*b*). Four ossified ceratobranchials are present. The first three are slender and the fourth is substantially more robust. Three short, poorly preserved hypobranchials lie near the front of the gill skeleton. Median endoskeletal components are unmineralized. However, this region is covered by an oval dental field composed of six abutting tooth plates, arranged in two anteroposteriorly directed rows of three plates each (figure 1*c*). It appears that the individual plates are serially associated with arches one through three. This large composite plate opposes the dental field of the overlying vomer and entopterygoids and similarly bears a single generation of small, pointed teeth. Additional isolated tooth plates may be associated with other parts of the branchial skeleton. Mineralization of the dorsal skeleton is restricted to possible pharyngobranchials.

### (c) Mandible

The dentary constitutes most of the outer face of the slender mandible (figure 1*f*,*g*). It bears the mandibular sensory canal, prominent tuberculate external ornament and a row of small conical teeth. An angular and possible surangular, plus a displaced articular, make up the posterior end of the jaw. The inner face of the mandible is lined with an indeterminate number of coronoids plus a lozenge-shaped prearticular that bears a lateral process defining the anterior margin of the adductor fossa. These inner bones bear very fine denticles that are beyond the limits of segmentation.

## 4. Discussion

### (a) Evolution of a tongue-bite mechanism in Palaeozoic actinopterygians

**Figure 3.**
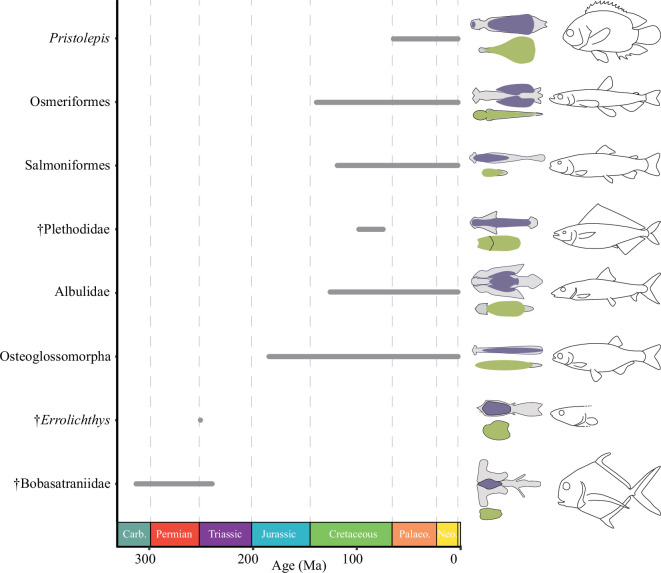
Multiple origins of tongue-bite mechanisms in actinopterygians. Stratigraphic range (left), schematic drawing of tongue bite components (middle) and body profile (right) of actinopterygian lineages with tongue bites. Ranges for extinct taxa are based on fossil occurrences; ranges for extant taxa are based on molecular clock estimates of total-group ages from [20,21]. Colour coding: grey, midline supporting skeletal elements; purple, upper dentigerous region; yellow, lower dentigerous region. Tongue bite schematics based on CT data (this article; electronic supplementary material, table S1) and [22–24]. Body profile schematics of extant taxa adapted from [25] and of extinct taxa adapted from [23,24,26,27].

Our three-dimensional tomographic data for †*P. parvulus* provide the earliest evidence of a tongue bite in actinopterygians (figure 3). Other early examples come from younger bobasatraniids (figure 2): the Late Pennsylvanian (Kasimovian; approx. 305 Ma) †*‘P.*’ *schultzei* bears an expanded vomerine tooth plate plus a single ventral one [28], with a similar, but more substantial, apparatus apparent in three-dimensional Early Triassic material of †*Bobasatrania mahavavica* and †*Bobasatrania groenlandica* [26]. Although *in situ* tooth plates are rare, abundant occurrences of isolated examples are known from the Late Pennsylvanian to the Middle Triassic [28–30].

The tooth plates of the older †*P. parvulus* correspond positionally to those of †bobasatraniids, but there are important structural differences (figure 2). First, the vomerine plate is narrow and relatively flat rather than broad and convex in younger taxa. Second, the faintly convex basibranchial plate is composed of two rows of three separate but tightly adjoining plates, in contrast to a single massive plate in †bobasatraniids. Third and finally, both the upper and lower plates of †*P. parvulus* bear only a single layer of pointed cusps, in contrast to the multiple superposed generations seen in bobasatraniids (so-called ‘phyllodont’ plates [29,31]). For all three features, the condition in †*P. parvulus* appears plesiomorphic relative to †bobasatraniids. Branchial tooth plates that are small, flat and bear a single layer of teeth or denticles are widespread among early osteichthyans, with basibranchial tooth plates typically consisting of paired rather than median bones [32].

The plesiomorphic construction of dental plates of †*P. parvulus* suggests a role in the evolution of the more elaborate tongue bites of †bobasatraniids. A close relationship between at least some †platysomids and †bobasatraniids has previously been proposed [9,11,19,28,29,33]. We find additional anatomical support for this hypothesis not only from the tongue bite but also by the highly unusual enclosure of the spiracular canal within the body of the ascending process of the parasphenoid. However, †*P. parvulus* lies outside of †bobasatraniids (*sensu* Schultze *et al.* [19]) based on retained plesiomorphic features found in generalized early actinopterygians (e.g. †*Raynerius splendens*: figure 2; [16]), including the absence of a highly modified quadratojugal and the presence of long, tooth-bearing jaws (figure 1*f*,*g*) rather than short edentulous ones (electronic supplementary material, figure S5). These primitive mandibular traits suggest that †*P. parvulus* retained use of the jaws in biting, with additional prey manipulation and reduction provided by the basibranchial dental plates. This stands in contrast to what seems to be the exclusive use of a tongue bite in †bobasatraniids, with the toothless jaws—which may have been kinetic in some species [26,34]—instead dedicated to governing fluid flow into the oral chamber. It is possible that shifting food processing to the branchial skeleton lifted constraints on the mandibular jaws in later †bobasatraniids, allowing them to become more mobile. Therefore †*P. parvulus* captures a key intermediate stage for one of the most specialized feeding mechanisms to evolve among Palaeozoic actinopterygians.

### (b) Tongue bites as a recurrent and versatile functional innovation

Tongue bites have evolved numerous times in actinopterygian lineages, including in several entirely extinct groups [32], with substantial variation in the number of tooth-bearing palatal and branchial elements and the extent of the dentigerous covering (figure 3) [35–37]. Although principally applied to the mechanism in osteoglossomorphs (bony tongues), there has been widespread use of the term in the literature. Here, we broadly define a tongue bite as the use of midline elements of the gill skeleton opposing toothed surfaces on the roof of the mouth. Other lineages, like galaxiiforms and argentiniforms, display prominent ventral midline dentition on the anterior gill elements but appear to lack—or have lost—the dorsal dentigerous elements of a tongue bite [37]. The tongue bite seen in †*P. parvulus* and later †bobasatraniids is the earliest known example of this mechanism in actinopterygians, which has evolved numerous times, including in several entirely extinct groups. The enigmatic Early Triassic †*Errolichthys* represents the next oldest case of an actinopterygian tongue bite, with a large oval basihyal dental plate opposing a similar field on the parasphenoid [22,23]. Late Cretaceous †plethodids represent the most diverse extinct clade characterized by a tongue bite, with nearly 20 genera known from this early diverging crown teleost lineage [24]. These are joined by additional isolated examples in Mesozoic teleost groups (e.g. †*Cimolichthys*; [38]).

The tongue-bite mechanism of living teleosts provides an actualistic model for interpreting function in †*P. parvulus* and †bobasatraniids. Osteoglossomorphs are the most celebrated extant tongue-biting group, with many species bearing prominent fangs on the parasphenoid, basihyal or both [3]. A tongue bite involving fangs is also present in salmoniforms, with *in vivo* kinematic studies of both groups showing prey reduction via ‘raking’ [4,39]. Tongue bites incorporating low crowns rather than long pointed teeth are better structural—and presumably functional—models for examples in Palaeozoic actinopterygians. In *Pristolepis* (leaffish), the basihyal plate and parasphenoid have opposing fields of rounded teeth [40]. Along with additional adjacent pharyngeal tooth plates, these contribute to a complex chewing cycle [40] for breaking down the cuticle of insects and insect larvae [41]. *Albula* (bonefish) provides the best analogue for Palaeozoic tongue biters. It possesses phyllodont basibranchial and parasphenoid plates with robust, rounded teeth superposed in several layers, with a rich fossil record of these phyllodont plates extending into the Cretaceous [31]. By weight, *Albula* stomach contents are dominated by hard prey like crustaceans and molluscs [42]. This suggests durophagy in †bobasatraniids, further supported by wear on the dental plates of †*B. scutata* [30].

### (c) Tongue bites as a component of the early trophic radiation of actinopterygians

Actinopterygians show substantial expansion in taxonomic and morphological diversity in the Carboniferous relative to the Devonian [8,43]. Past work suggests this shift might relate to diversification spurred by ecological opportunities opened by the Devonian–Carboniferous extinction [44,45]. In particular, the Carboniferous records a proliferation of tooth and jaw traits bearing on feeding ecology. These include the appearance of coronoid processes (several taxa; [46,47]); styliform (†Guildayichthyiformes [48]), peg-like (†*Frederichthys* [49]), and bulbous (†*Mesolepis* [50]) teeth; strongly heterodont dentitions (†*Paphosiscus* [51]); large fangs (several potentially unrelated lineages; [14]); and dense dental batteries, consolidated dental plates and beaks (†Eurynotiformes; [9,10]). Many represent the first examples of traits that would go on to appear independently in later, distantly related ray-fin lineages.

Tongue bites represent an additional feeding innovation in Carboniferous actinopterygians, but their first appearance postdates many other examples. The oldest †platysomids preserving cranial material date to the mid-Mississippian (mid-Viséan; approx, 338 Ma [52]) and show no evidence of opposing dorsal and ventral dental plates, but the arrangement in the younger †*P. parvulus* indicates it might not be long removed from the origin of the tongue bite itself. However, the multipartite construction of its primitive dorsal and ventral plates suggests that isolated components of early tongue bites might not be found or recognized, so we cannot exclude an earlier origin. This contrasts with the relatively continuous record of phyllodont plates of related †bobasatraniids in Upper Pennsylvanian to Middle Triassic deposits, representing a duration of approximately 70 Myr for this successful lineage of hard-prey specialists. A delayed origin of a crushing tongue bite relative to the Devonian–Carboniferous transition differs from the pattern for the other principal lineage of durophagous Palaeozoic actinopterygians: †eurynotiforms. The earliest †eurynotiforms already show specializations for consuming hard prey in the Tournaisian [9]. By the mid-Viséan, taxa like †*Cheirodopsis* possess highly consolidated upper and lower tooth plates and beak-like oral jaws [52]. The offset origins of these different anatomical solutions to the shared problem of processing hard prey reinforces other evidence for a protracted interval of innovation in early actinopterygians, spanning the Carboniferous and potentially extending back to the latest Devonian [53,54]. Ultimately, placing these functional traits within an explicitly phylogenetic context will be necessary for testing whether feeding innovations arose at a steady rate or instead show an evolutionary burst consistent with ecological release.

## Data Availability

Reconstructed tomogram stacks (as .TIFF files) and surface meshes for three-dimensional segmented elements (as .PLY and .OBJ files) of specimens scanned for this study are archived on MorphoSource and Zenodo. Full details are given in the electronic supplementary material. Supplementary material is available online [55].
